# Tc-99m DMSA Radiomics in CKD: Phenotype-Specific Cortical Signatures and a Morphological Predictor of Renal Function Decline

**DOI:** 10.3390/diagnostics16091351

**Published:** 2026-04-30

**Authors:** Mustafa Demir, Nihat Köylüce, Davut Eren, Koray Uludağ, Hümeyra Gençer, Seyhan Karaçavuş, Fadime Demir

**Affiliations:** 1 Nephrology Department, Kayseri City Hospital, Şeker Mah. Muhsin Yazicioglu Bulvarı No:77, Kocasinan, 38080 Kayseri, Türkiye; dr.davuteren@gmail.com (D.E.); kuludag@gmail.com (K.U.); 2 Nuclear Medicine Department, Kayseri City Hospital, Şeker Mah. Muhsin Yazicioglu Bulvarı No:77, Kocasinan, 38080 Kayseri, Türkiye; nhtkylc91@gmail.com (N.K.); drhumeyra@yahoo.com (H.G.); seyhankaracavus@hotmail.com (S.K.); drfadimedemir@hotmail.com (F.D.)

**Keywords:** chronic kidney disease, imaging phenotypes, radiomics, renal cortical uptake, Tc-99m DMSA scintigraphy

## Abstract

**Purpose**: This study aims to evaluate the ability of radiomic features obtained from technetium-99m dimercaptosuccinic acid (Tc-99m DMSA) planar images to distinguish renal cortical uptake patterns among patients with chronic kidney disease (CKD). We also assessed the association between selected radiomic features and progressive renal function loss during follow-up. **Methods**: The study included a total of 185 patients: patients with Diabetes mellitus (DM) + hypertension (HTN) diagnosis (Group 1, n = 30), patients with HTN diagnosis alone (Group 2, n = 86), and patients with no history of DM or HTN who were followed for CKD (Group 3, n = 69). Intergroup comparisons were performed using the Kruskal–Wallis test with Bonferroni-corrected post hoc pairwise testing; the proportion of significantly different features was assessed using FDR correction. As a secondary exploratory analysis, the relationship between selected radiomic features and time to first observed ≥20% eGFR decline at follow-up was evaluated using univariate L2-penalised Cox proportional hazards regression with feature selection guided by the events-per-variable principle and model discrimination quantified using Harrell’s C-index. **Results**: Intensity Kurtosis values showed a statistically significant difference among the groups: −0.11 (−0.31 to 0.12) for Group 1, −0.24 (−0.41 to −0.04) for Group 2, and −0.33 (−0.45 to −0.16) for Group 3 (*p* = 0.001). Mean Intensity values were found to be 60.66 (31.01–89.39) in Group 1 and 90.46 (72.87–106.34) in Group 3 (*p* < 0.001). Age, gender, and baseline eGFR did not differ between groups. Radiomic analysis revealed significant intergroup differences predominantly in intensity- and texture-based features, while morphological features showed more limited differentiation. In the secondary exploratory longitudinal analysis, Centre of Mass Shift was the only morphological feature significantly associated with time to first observed ≥20% eGFR decline at follow-up (HR per SD: 0.74; 95% CI: 0.58–0.94; *p* = 0.015; C-index: 0.57). **Conclusions**: Radiomic features from Tc-99m DMSA planar images reveal quantitative differences between clinically defined CKD subgroups even when cortical uptake appears visually indistinguishable. The threshold-specific association of Centre of Mass Shift with subsequent eGFR decline, beyond baseline renal function, suggests that DMSA radiomics may provide exploratory prognostic information that warrants prospective validation.

## 1. Introduction

Chronic kidney disease (CKD) is a condition frequently encountered in clinical practice that can lead to serious morbidities [1]. The clinical importance of CKD is also reflected in the growing burden of kidney replacement therapy across Europe [2]. Beyond Europe, CKD is also a major global health burden and is projected to become one of the leading causes of years of life lost worldwide by 2040 [3]. Disease management in daily practice is generally based on estimated glomerular filtration rate (eGFR) and urinary albumin-to-creatinine ratio (uACR). Although these parameters are clinically essential, their primary function is to reflect overall renal function. In contrast, structural and functional changes at the parenchymal level may begin before a significant decline in eGFR occurs. Especially in early-stage CKD, this situation causes a time lag between tissue damage and clinically measurable loss of function [4].

Diabetes mellitus (DM) and hypertension (HTN) are two of the most common causes of CKD [1]. In clinical practice, diagnosis of DM or HTN does not always indicate that kidney involvement is directly related to these etiologies. Especially in the early stages when renal function is largely preserved, it can be difficult to distinguish the underlying cause of kidney damage based on clinical and laboratory findings [5,6]. Although kidney biopsy remains the reference method for etiological clarification, current guidelines recommend a selective rather than routine approach based on the clinical context and level of suspicion [4]. At the same time, its invasive nature and the risk of procedure-related complications, particularly bleeding, limit its role as a routine tool in broader early-stage evaluation [7]. This situation highlights the importance of non-invasive and additional evaluation methods that can reflect tissue-level differences in early-stage patients.

Radiomics is an approach that aims to extract quantitative features from medical images that cannot be distinguished by the human eye and to analyze these features in a way that reflects tissue characteristics. Features such as density distribution, spatial heterogeneity, and tissue organization enable images to be converted into a numerical representation of tissue [8,9]. Radiomics studies in the field of CKD have mostly been conducted using cross-sectional imaging methods, and it has been shown that some radiomic features may be related to renal structure and disease heterogeneity [10,11]. However, the use of radiomics in functional nuclear medicine imaging is still limited, with most of the available literature centred on PET and predominantly on oncologic applications, leaving comparatively less experience in renal scintigraphy [12].

Technetium-99m dimercaptosuccinic acid (Tc-99m DMSA) scintigraphy is a well-established imaging modality for the assessment of renal cortical function and parenchymal integrity. The preferential binding of Tc-99m DMSA to the renal cortex allows the acquired images to reflect viable cortical parenchyma with a high degree of specificity [13,14]. In clinical practice, the interpretation of Tc-99m DMSA scintigraphy largely relies on qualitative assessment, primarily aimed at identifying overt cortical defects or marked interrenal asymmetry [13]. Subtle regional differences in cortical uptake, however, are often not readily appreciated on visual analysis. In this context, quantitative analysis of planar Tc-99m DMSA images may provide a more refined assessment of intrarenal functional heterogeneity [15].

This study aimed to evaluate whether radiomic features derived from Tc-99m DMSA images can distinguish renal parenchymal patterns across clinically defined CKD subgroups. We further explored whether these radiomic patterns are associated with clinically relevant renal function loss during follow-up.

## 2. Material and Methods

This retrospective, single-centre study included patients with chronic kidney disease who were followed in the nephrology clinic between 2018 and 2025 and underwent Tc-99m DMSA scans during follow-ups. The study was approved by the Non-Interventional Clinical Research Ethics Committee of Kayseri City Hospital (Decision No: 523, Date: 8 May 2025).

All included patients met the KDIGO-based clinical definition of CKD, as documented in the nephrology follow-up records. Group 1 included patients with a documented history of both diabetes mellitus and hypertension who had received treatment, including lifestyle measures and/or medical therapy. Group 2 included patients with a documented history of hypertension without diabetes who had received treatment. Group 3 included patients with CKD but no documented history of either diabetes mellitus or hypertension and was therefore considered a heterogeneous non-DM/non-HTN CKD subgroup. Urinary albumin-to-creatinine ratio (uACR) and urinary albumin-to-protein ratio (uPCR) were recorded separately.

Baseline clinical and laboratory variables were retrieved from the hospital information system. Serum creatinine was measured in the central laboratory using an enzymatic method, and eGFR values recorded in the system were calculated using the CKD-EPI 2021 equation.

Images were obtained according to standard protocols. Analyses were performed on posterior projection planar images. Region of interest (ROI) delineation for both kidneys in posterior projection Tc-99m DMSA images was performed using a semi-automatic thresholding method. ROIs were created to include intensity values at least 40% of the highest pixel intensity in the image. This thresholding defined functionally active cortical areas and limited the effect of low-intensity background. ROIs were defined separately for the right and left kidneys. Radiomic feature extraction was performed on the defined ROIs using the LIFEx (Local Image Features Extraction) software version v7.8.0 [16]. Given the planar nature of the images, a 2D approach was used for the analysis, employing the software’s 2DCo-occurrence (2DCor) option.

The following parameters were used for intensity segmentation in radiomic analyses:

• Number of gray levels: 64
• Size of bins: 0.3125
• Minimum bound: 0.0
• Maximum bound: 20.0

These settings were used to ensure consistent intensity discretisation across cases and to improve the comparability of the extracted radiomic features. Morphological features, intensity-based features, density histogram-based features, and Gray Level Run Length Matrix (GLRLM)-based texture features were extracted from each kidney ROI as parameters belonging to radiomic feature groups. All radiomic features were extracted using the default computational framework implemented in LIFEx software.

The radiomic parameters obtained from each kidney were scaled using a functional integration approach with the split renal function value of the corresponding kidney. The weighted values obtained for the right and left kidneys were then summed and normalized by dividing by 100. This method was used to obtain composite radiomic values reflecting bilateral renal contribution on an individual basis.

The chi-square test was used for comparisons between groups of categorical variables. Continuous variables are given as median [interquartile range]. The Kruskal–Wallis test was used for comparisons of radiomic features obtained from Tc-99m DMSA scintigraphy between groups. Post hoc pairwise analyses were performed with Bonferroni correction for variables found to be statistically significant. To characterise the overall proportion of features showing intergroup differences, false discovery rate (FDR) correction was applied using the Benjamini–Hochberg method across all 65 radiomic features; effect sizes were quantified as rank-biserial correlation coefficients (r).

As a secondary and exploratory analysis, the association between selected radiomic features and time to renal function loss was examined. The event endpoint was defined as the first occurrence of a ≥20% decline in eGFR from baseline at any scheduled follow-up visit. Of the 185 patients, 176 (44 events) were eligible for Cox analyses; 9 patients were excluded due to missing follow-up time data. Given right-censored data and variable follow-up durations, time-to-event analysis was performed using univariate L2-penalised Cox proportional hazards regression (Ridge penaliser λ = 0.1). All radiomic variables were standardised to z-scores prior to modelling. Feature selection was guided by the events-per-variable (EPV) principle to maintain EPV ≥ 10; specifically, features were selected a priori to represent distinct aspects of renal cortical structure: morphological asymmetry (Centre of Mass Shift), renal volume (Voxels Counting and Surface Area, with the latter excluded from the adjusted model due to near-collinearity with Voxels Counting, r = 0.96), and cortical signal intensity (RMS Intensity). Model discrimination was quantified using Harrell’s concordance index (C-index). Threshold-sensitivity analyses were conducted at ≥10% and ≥30% eGFR decline definitions. A two-variable adjusted model was fitted combining Centre of Mass Shift and Voxels Counting (EPV = 22.0).

Statistical analysis of radiomic measurements was performed using Python 3.13.5 (Python Software Foundation, Beaverton, OR, USA) and scikit-learn 1.8.0.. Descriptive and regression analyses of clinical variables were performed using the IBM SPSS Statistics 22.0 (IBM Corp., Armonk, NY, USA) software package. In all statistical tests, a two-tailed *p* < 0.05 value was considered statistically significant. This study was reported in accordance with the STROBE guidelines.

## 3. Results

Patient characteristics

The study included a total of 185 patients: 30 in Group 1, 86 in Group 2, and 69 in Group 3. The median age in Group 1, Group 2, and Group 3 was 49.0 (40.2–57.0), 44.5 (37.2–50.0), and 43.0 (36.0–48.0) years, respectively, with no statistically significant difference between groups (*p* = 0.071). Female gender ratios were similar across groups (*p* = 0.377). Baseline eGFR value was 86.5 (58.8–110.2), 84.5 (60.0–105.8), and 81.0 (72.0–109.0) mL/min/1.73 m^2^ in Group 1, 2, and 3, respectively, and did not differ significantly (*p* = 0.640). In contrast, uPCR levels showed significant differences between groups; the highest values were observed in Group 1 [170.6 (104.9–804.5) mg/g] (*p* < 0.001). Angiotensin-converting enzyme inhibitor/angiotensin receptor blocker (ACEi/ARB) use showed a significant difference between groups and was detected at a higher rate in Group 1 (76.7%) and Group 2 (95.3%) compared to Group 3 (29.0%) (*p* < 0.001). No statistically significant differences were observed between the groups in terms of other clinical and laboratory variables. Detailed demographic and clinical characteristics of the patients are presented in Table 1.

Radiomic differences across clinically defined CKD subgroups

In the analysis of radiomic features obtained from the Tc-99m DMSA scintigraphy, statistically significant differences were observed between the groups in multiple quantitative radiomic parameters, despite the absence of a distinct cortical defect or uptake pattern in the visual assessment of planar images. Significant differences were predominantly observed in intensity-based and texture-based radiomic features, while fewer differences were noted in morphological radiomic features. Full feature-level results are shown in Supplementary Table S1.

Intensity Kurtosis values showed a statistically significant difference among the groups: −0.11 (−0.31 to 0.12) for Group 1, −0.24 (−0.41 to −0.04) for Group 2, and −0.33 (−0.45 to −0.16) for Group 3 (*p* = 0.001). Mean Intensity values were found to be 60.66 (31.01–89.39) in Group 1 and 90.46 (72.87–106.34) in Group 3 (*p* < 0.001). The Intensity Variance parameter was 162.69 (48.37–299.96) in Group 1, 273.42 (145.81–402.77) in Group 2, and 359.91 (215.35–476.91) in Group 3 (*p* < 0.001); the lower variance in Group 1 is discussed in the context of GLRLM texture findings below. Other significant radiomic features based on the intensity histogram are presented in Table 2.

The Long Runs Emphasis values from the GLRLM-based texture parameters were measured as 55.38 (21.07–83.72) in Group 1, 78.49 (56.63–92.02) in Group 2, and 84.55 (57.93–100.77) in Group 3 (*p* = 0.005). Run Percentage values also showed statistically significant difference between groups and were determined as 0.73 (0.72–0.77) in Group 1, 0.72 (0.69–0.75) in Group 2, and 0.71 (0.69–0.74) in Group 3 (*p* = 0.001). Other significant radiomic parameters based on texture are presented in Table 3.

In terms of morphological radiomic features, statistically significant differences were detected between groups in the Compacity, Spherical Disproportion, Asphericity, and Centre of Mass Shift parameters (*p* < 0.05). These differences were primarily observed in comparisons between Group 1 and Group 3. Other significant morphological radiomic features are presented in Table 4.

In pairwise comparisons between groups, most of the significant differences were observed in comparisons between Group 1 and Group 3. For example, while the Group 1–Group 3 comparison was statistically significant for Median Intensity (*p* < 0.001), the difference in the Group 2–Group 3 comparison was more limited. Similar patterns were observed for the Intensity Variance and Long Runs Emphasis parameters. Detailed results for all pairwise comparisons are presented in Table 2, Table 3 and Table 4.

Exploratory analysis of radiomic features and renal function loss

**Figure 1:**
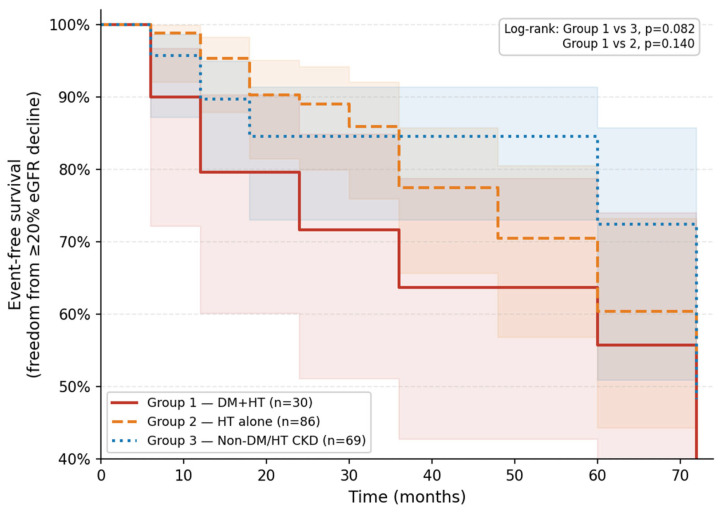
Kaplan–Meier curves for freedom from ≥20% eGFR decline. Event-free survival curves are shown for Group 1 (DM + HTN; solid line), Group 2 (HTN alone; dashed line), and Group 3 (non-DM/HTN CKD; dotted line). The endpoint was defined as the first occurrence of a ≥20% decline in eGFR from baseline at any scheduled follow-up visit (n = 49 events total; 26.5%). Log-rank *p* values for pairwise comparisons are annotated in the figure. Median follow-up: 36 months (IQR 24–60). eGFR, estimated glomerular filtration rate; DM, diabetes mellitus; HTN, hypertension; CKD, chronic kidney disease.

**Figure 2:**
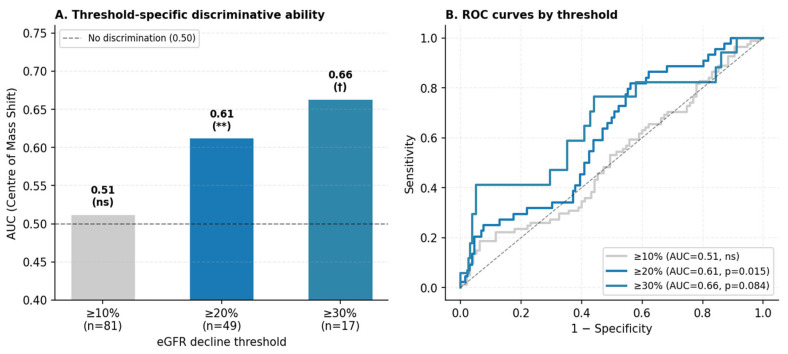
*Threshold-specific discriminative ability of Centre of Mass Shift for eGFR decline.* (**A**): AUC values from receiver operating characteristic analysis at ≥10% (n = 81 events), ≥20% (n = 49), and ≥30% (n = 17) eGFR decline thresholds. Significance annotations: ** *p* < 0.05; † *p* < 0.10; ns, not significant. (**B**): corresponding ROC curves. The progressive increase in AUC across more stringent thresholds indicates that Centre of Mass Shift specifically discriminates progressive, clinically meaningful decline rather than transient eGFR fluctuations. AUC, area under the receiver operating characteristic curve.

Using the any-visit event definition, a first occurrence of ≥20% eGFR decline from baseline was identified in 49 of the 185 patients (26.5%) over a median follow-up of 36 months (IQR 24–60). Event rates were 43.3% in Group 1 (n = 13), 26.7% in Group 2 (n = 23), and 18.8% in Group 3 (n = 13). Of note, Group 1 also recorded the lowest median Centre of Mass Shift value across groups (1.14 vs. 1.28 in Group 2 and 1.23 in Group 3; Table 4), consistent with the direction of the Cox association reported below. Kaplan–Meier curves are presented in Figure 1; log-rank testing did not demonstrate statistically significant between-group differences (Group 1 vs. Group 3, *p* = 0.082; Group 1 vs Group 2, *p* = 0.140). In univariate L2-penalised Cox regression (Table 5), Centre of Mass Shift was the only morphological feature reaching statistical significance (HR per SD increase: 0.74; 95% CI: 0.58–0.94; *p* = 0.015; C-index: 0.57). Lower values of this parameter—reflecting reduced displacement of the intensity—weighted from the geometric centroid were associated with faster eGFR decline. Surface Area and Voxels Counting also demonstrated significant univariate associations (HR: 0.68 [0.52–0.88], *p* = 0.003 and HR: 0.67 [0.52–0.87], *p* = 0.003, respectively), though near-collinearity between the two (r = 0.96) precludes independent interpretation. RMS Intensity was not significantly associated with the outcome (HR: 0.92; *p* = 0.497). In a two-variable adjusted model combining Centre of Mass Shift and Voxels Counting (EPV = 22.0; C-index: 0.641), Centre of Mass Shift retained a consistent directional effect (HR: 0.79; 95% CI: 0.62–1.02; *p* = 0.067), while Voxels Counting reached statistical significance (HR: 0.71; 95% CI: 0.55–0.93; *p* = 0.011); the borderline significance of Centre of Mass Shift in the adjusted setting likely reflects reduced statistical power rather than loss of the underlying signal. In a post hoc sensitivity analysis adjusting for ACEi/ARB use and baseline uPCR (n = 161; 40 events), the association of Centre of Mass Shift with eGFR decline remained essentially unchanged (HR: 0.71; 95% CI: 0.55–0.93; *p* = 0.014), indicating that its prognostic signal is independent of these clinical factors. In threshold-sensitivity analyses, Centre of Mass Shift showed no discrimination at ≥10% (AUC: 0.51; *p* = 0.317), but discriminative ability increased progressively for ≥20% (AUC: 0.61; *p* = 0.015) and ≥30% decline (AUC: 0.66; *p* = 0.084), as shown in Figure 2.

## 4. Discussion

This study shows that even in cases where no obvious cortical defect or clear uptake pattern was observed in the visual assessment of posterior planar Tc-99m DMSA images, clinically meaningful group distinctions could be identified through radiomic analysis. Tc-99m DMSA images that appear visually similar in patients with DM and HTN comorbidity and in CKD patients without a history of DM/HTN nonetheless exhibit quantitatively distinct patterns.

There was no significant difference between groups in terms of age, gender, and baseline eGFR levels; yet, the distinct differentiation of Tc-99m DMSA radiomic features, particularly in the DM + HTN group, warrants consideration. These radiomic differences were observed despite the high use of ACEi/ARB in Groups 1 and 2, suggesting that treatment exposure alone is unlikely to account for the overall pattern. However, the marked imbalance in ACEi/ARB use across groups complicates interpretation, and treatment-related effects on cortical uptake cannot be fully excluded. Baseline eGFR was largely preserved across groups, suggesting that the observed radiomic differences were not merely driven by overt renal dysfunction. These features suggest that the radiomic differences observed are unlikely to represent end-stage parenchymal injury and may instead reflect early, subclinical cortical changes. In fact, there are reports in the literature emphasizing that Tc-99m DMSA cortical uptake may be related to proximal tubular function and live cortical cell mass, in a manner that is at least partially independent of glomerular filtration rate [15,17]. Taken together, these visually indistinguishable yet radiomically detectable differences suggest that functional cortical heterogeneity may be quantitatively captured at an early stage.

The significance of intensity-based parameters—particularly the full range of intensity-level features (Mean, Median, percentile values, Standard Deviation)—indicates that the DM + HTN group exhibits uniformly lower overall cortical uptake across all intensity metrics. Importantly, Intensity Variance is also lower in Group 1 (162.69) than in Groups 2 (275.08) and 3 (359.91). This is not a sign of uniformity in the usual sense; rather, it reflects a downward compression of the signal: the DM + HTN group’s cortical activity operates within a narrower and lower intensity range, consistent with diffuse parenchymal involvement that leaves little room for pixel-level variability. The texture domain adds a distinct layer to this picture. GLRLM-based features (Short Runs Emphasis, Long Runs Emphasis, Run Percentage) reveal that gray-level values within the DM + HTN ROI form shorter and less coherent sequential runs. This pattern—higher Short Runs Emphasis and lower Long Runs Emphasis in Group 1—reflects irregular spatial organisation of cortical activity, a form of heterogeneity that intensity statistics, precisely because they are reduced and compressed, cannot express on their own. Together, the two feature families describe different facets of the same underlying pathology: globally reduced and spatially disorganised cortical uptake. The HTN-only group occupies an intermediate position in most intensity and texture parameters, suggesting that DM contributes an additional parenchymal burden beyond what hypertension alone produces [18,19].

Limited significance of morphological radiomic features is consistent with the two-dimensional nature of planar Tc-99m DMSA images and the ROI-based analysis approach [20]. Shape and boundary-based criteria may be more sensitive to the segmentation definition in planar projections, which may limit the distinctive contribution of these features [21]. The dominant role of intensity and texture-based features in group differentiation suggests that cortical fixation patterns differ more in terms of activity distribution and spatial organization than in terms of shape. Accordingly, morphological features appear to function as secondary components supporting the overall radiomic pattern rather than as primary differentiating axes.

The radiomic patterns presented in this study suggest that Tc-99m DMSA planar images carry a more refined and phenotype-sensitive layer of information than is accessible through global functional assessment or the detection of overt cortical defects. These findings may be viewed, within the concept of a “digital biopsy,” non-invasive quantitative characterisation of cortical uptake patterns that reveals phenotypic distinctions without implying histopathological equivalence [8,9]. In this sense, the cross-sectional findings support a phenotyping role that is distinct from, but complementary to, the exploratory longitudinal analysis.

The secondary longitudinal analysis identified Centre of Mass Shift as a morphological feature independently associated with progressive eGFR decline. This parameter captures the spatial displacement between the geometric centroid of the renal ROI and the intensity-weighted centroid; a larger shift indicates that peak cortical activity is distributed asymmetrically relative to the overall uptake area. Because this parameter was derived from planar posterior projections, part of the measured displacement may also reflect projection-related effects rather than intrarenal asymmetry alone. This observation also carries internal consistency across the study’s two analytical components: Group 1, which had the lowest median Centre of Mass Shift at baseline (1.14), was the same group with the highest subsequent event rate (43.3%). While this cross-sectional–longitudinal concordance does not establish causality, it strengthens the plausibility of the association. A further strength is the negligible Spearman correlation between Centre of Mass Shift and baseline eGFR (ρ = 0.07), confirming that its predictive signal is independent of current renal function and kidney volume. This independence extended to established clinical risk factors: in a post hoc sensitivity analysis additionally adjusting for ACEi/ARB use and baseline uPCR, the effect estimate was materially unchanged (HR: 0.71), suggesting that Centre of Mass Shift captures prognostic information beyond what is reflected in treatment intensity or proteinuric burden. This distinguishes it from Surface Area and Voxels Counting, which correlated more substantially with baseline eGFR (ρ ≈ 0.41 and 0.39, respectively) and likely reflect the established relationship between renal mass and functional reserve [22]. The two-variable adjusted model indicates that Centre of Mass Shift and Voxels Counting provide at least partially non-overlapping prognostic information. The threshold-sensitivity analyses add an important interpretive dimension. Centre of Mass Shift did not discriminate ≥10% eGFR decline (AUC: 0.51), a threshold that captures haemodynamic fluctuations alongside true progression [23]. Discriminative ability increased at the ≥20% and ≥30% thresholds, suggesting that reduced spatial variation in cortical uptake may be more closely related to clinically meaningful renal function decline than to minor short-term fluctuations [24]. A lower Centre of Mass Shift may reflect a more diffusely reduced cortical activity pattern, whereas a larger shift may indicate greater regional variation in preserved uptake, although this interpretation remains speculative [25,26]. This interpretation is consistent with the cross-sectional observation that Group 1, which exhibited the most diffuse uptake reduction across intensity parameters, also carried the lowest Centre of Mass Shift. The C-index values of 0.57 (univariate) and 0.64 (adjusted model) reflect modest but non-trivial discrimination, appropriate for a single imaging biomarker in a heterogeneous CKD cohort. These observations require prospective validation before any clinical utility can be claimed.

Several limitations merit consideration. The retrospective single-centre design constrains external generalisability. The relatively small size of Group 1, together with the imbalance across groups, may have reduced the precision of pairwise comparisons and supports a cautious interpretation of the findings. Radiomic analyses were performed on planar posterior projections, which do not capture the full three-dimensional spatial heterogeneity of the kidney and may be influenced by projection-related factors; SPECT-based extraction may yield richer feature sets in future studies. The semi-automatic 40% thresholding approach is reproducible but does not account for interpatient variation in background activity or body habitus. The aetiological heterogeneity of Group 3—encompassing a range of non-DM, non-HTN diagnoses—limits the precision of comparative inference and may attenuate true effect sizes. In the longitudinal analysis defining the event as the first observed decline at follow-up may have captured some transient eGFR reductions related to haemodynamic changes or intercurrent illness rather than true disease progression. A confirmed sustained-decline definition would strengthen causal inference but was not feasible with the available follow-up structure. The radiomic signatures have not been externally validated, and prospective replication is necessary before any prognostic application can be considered.

This study demonstrates that radiomic features derived from Tc-99m DMSA planar images reveal quantitative differences between CKD phenotypes even when cortical uptake appears visually indistinguishable. The differentiation of the DM + HTN group from others was consistent across intensity, texture, and selected morphological feature classes, with effect sizes indicating moderate to large between-group separation. Beyond cross-sectional phenotyping, the secondary analysis introduces Centre of Mass Shift as a morphological marker with threshold-specific prognostic behaviour that is independent of baseline renal function and provides complementary information to purely volumetric features. Taken together, these findings suggest that quantitative DMSA scintigraphy offers information layers not accessible through conventional visual interpretation or standard functional metrics. In larger, prospectively designed, and externally validated studies, the role of DMSA radiomics in cortical characterisation and progression monitoring could be more rigorously defined.

## Figures and Tables

**Table 1:** Baseline Demographic and Clinical Characteristics.

Variable	Group 1 (DM + HTN) (*n* = 30)	Group 2 (HTN) (*n* = 86)	Group 3 (Non-DM/Non-HTN CKD) (*n* = 69)	*p* Value
Age (years)	49.0 (40.2–57.0)	44.5 (37.2–50.0)	43.0 (36.0–48.0)	0.071
Gender (Female/Male)	F: 18/M: 12	F: 44/M: 42	F: 31/M: 38	0.377
Cardiovascular Disease	4 (13.3%)	9 (10.5%)	1 (1.4%)	0.046
ACEi/ARB Usage	23 (76.7%)	82 (95.3%)	20 (29.0%)	<0.001
Baseline eGFR (mL/min)	86.5 (58.8–110.2)	84.5 (60.0–105.8)	81.0 (72.0–109.0)	0.640
Serum Creatinine (mg/dL)	0.9 (0.6–1.2)	1.0 (0.8–1.3)	1.0 (0.7–1.2)	0.665
BUN (mg/dL)	14.1 (11.2–19.4)	14.3 (11.0–19.9)	13.0 (10.0–16.0)	0.146
Sodium (mEq/L)	139.0 (138.2–140.8)	139.5 (138.0–141.0)	140.0 (138.0–141.0)	0.403
Potassium (mEq/L)	4.6 (4.3–5.0)	4.4 (4.1–4.7)	4.4 (4.3–4.6)	0.118
Chloride (mEq/L)	102.0 (101.0–103.8)	102.5 (100.0–104.0)	103.0 (102.0–105.0)	0.079
Calcium (mg/dL)	9.5 (9.3–9.8)	9.5 (9.2–9.8)	9.4 (9.2–9.6)	0.540
Phosphorus (mg/dL)	3.5 (3.2–4.0)	3.4 (2.8–3.8)	3.2 (2.8–3.6)	0.166
Total Protein (g/dL)	72.8 (69.9–76.3)	72.3 (70.0–75.2)	72.7 (69.7–75.2)	0.639
Albumin (g/dL)	45.0 (42.2–46.8)	45.1 (44.0–48.0)	45.0 (43.0–47.0)	0.475
PTH (pg/mL)	46.5 (33.5–62.5)	47.0 (33.0–56.0)	41.0 (33.0–58.5)	0.729
uPCR (mg/g)	170.6 (104.9–804.5)	102.0 (64.9–340.9)	68.9 (47.8–177.0)	<0.001
uACR (mg/g)	10.6 (8.9–14.1)	14.6 (8.3–108.9)	7.0 (4.3–37.4)	0.260
Hemoglobin (g/dL)	13.8 (12.7–15.4)	14.6 (13.5–15.8)	14.3 (13.0–16.0)	0.225
WBC (10^3^/µL)	8.3 (6.9–9.6)	7.9 (6.7–9.2)	7.6 (6.4–8.8)	0.408
PLT (10^3^/µL)	277 (228–326)	281 (234–313)	273 (223–304)	0.641

Abbreviations: DM: Diabetes Mellitus; HTN: Hypertension; ACEi/ARB: Angiotensin-Converting Enzyme Inhibitors/Angiotensin Receptor Blockers; uACR: urinary albumin-to-creatinine ratio; uPCR: urinary protein-to-creatinine ratio; eGFR: Estimated Glomerular Filtration Rate; BUN: Blood Urea Nitrogen; WBC: White Blood Cell; PLT: Platelet; PTH: Parathyroid Hormone.

**Table 2:** Comparison of Tc-99m DMSA-based Radiomic Features: Intensity-Based Features.

Radiomic Feature	Group 1 (DM + HTN)	Group 2 (HTN)	Group 3 (Non-DM/Non-HTN CKD)	*p* Value	Post Hoc Analysis (*p* < 0.05)
Integrated Intensity	199,471 (109,509–307,559)	293,787 (220,058–332,885)	318,366 (270,730–362,739)	0.001	1–2, 1–3
Mean Intensity	60.66 (31.01–89.39)	79.27 (65.85–91.39)	90.46 (72.87–106.34)	<0.001	1–2, 1–3
Median Intensity	58.99 (31.11–86.92)	76.91 (63.93–89.83)	88.63 (69.78–103.19)	<0.001	1–2, 1–3
Minimum Intensity	41.30 (17.67–57.45)	54.43 (45.90–63.49)	61.17 (49.31–71.62)	<0.001	1–2, 1–3
Maximum Intensity	102.53 (56.14–142.21)	135.59 (114.93–157.18)	151.77 (122.40–177.67)	<0.001	1–2, 1–3
Intensity Variance	162.69 (48.37–299.96)	275.08 (179.47–379.94)	359.91 (215.35–476.91)	<0.001	1–2, 1–3
Intensity Kurtosis	−0.11 (−0.31–0.12)	−0.24 (−0.41–0.04)	−0.33 (−0.45–0.16)	0.001	1–3, 2–3
10th Intensity Percentile	45.14 (22.63–64.99)	59.90 (49.27–68.78)	68.09 (54.63–79.24)	<0.001	1–2, 1–3
25th Intensity Percentile	50.38 (25.96–75.63)	67.17 (55.05–76.60)	76.31 (60.79–88.45)	<0.001	1–3
75th Intensity Percentile	68.98 (35.93–100.07)	90.30 (74.82–104.53)	103.05 (82.53–121.07)	<0.001	1–2, 1–3
90th Intensity Percentile	78.59 (40.44–112.91)	102.32 (83.99–118.53)	117.85 (94.46–137.04)	<0.001	1–2, 1–3, 2–3
Standard Deviation	12.64 (6.91–17.09)	16.52 (13.18–19.35)	18.96 (14.67–21.84)	<0.001	1–2, 1–3
Intensity IQR	18.41 (9.81–24.53)	24.06 (18.71–29.19)	27.68 (20.99–31.89)	<0.001	1–2, 1–3
Intensity Range	61.22 (35.47–84.76)	81.11 (69.21–93.67)	90.63 (73.09–106.25)	<0.001	1–2, 1–3
Mean Absolute Deviation	10.31 (5.61–13.92)	13.55 (10.75–16.11)	15.40 (12.02–17.87)	<0.001	1–2, 1–3
Robust Mean Abs. Dev.	7.58 (4.16–10.53)	10.03 (7.90–12.20)	11.45 (8.84–13.30)	<0.001	1–2, 1–3
Median Absolute Deviation	10.21 (5.56–13.89)	13.41 (10.61–15.99)	15.26 (11.87–17.79)	<0.001	1–2, 1–3
Area Under Curve	19.17 (9.98–28.18)	25.01 (20.83–28.80)	28.49 (22.98–33.40)	<0.001	1–2, 1–3
Intensity Based Energy	3.1 M (969 k–6.6 M)	6.2 M (4.0 M–7.8 M)	7.5 M (5.2 M–9.6 M)	<0.001	1–3
Root Mean Square Intensity	61.97 (31.75–91.05)	80.90 (67.29–93.29)	92.46 (74.47–108.58)	<0.001	1–2, 1–3
Total Lesion Glycolysis	213.40 (123.95–324.97)	308.01 (234.68–349.11)	333.23 (288.09–383.33)	0.001	1–2, 1–3

**Table 3:** Comparison of Tc-99m DMSA -based Radiomic Features: Texture Features.

Radiomic Feature	Group 1 (DM + HTN)	Group 2 (HTN)	Group 3 (Non-DM/Non-HTN CKD)	*p* Value	Post Hoc Analysis (*p* < 0.05)
Short Runs Emphasis	0.79 (0.78–0.86)	0.78 (0.76–0.79)	0.77 (0.76–0.78)	<0.001	1-2, 1-3
Long Runs Emphasis	55.38 (21.07–83.72)	78.49 (56.63–92.02)	84.55 (57.93–100.77)	0.005	1-2, 1-3
Low Grey Level Run Emphasis	0.0002 (0.0002–0.0003)	0.0002 (0.0002–0.0002)	0.0002 (0.0002–0.0002)	0.001	1-2, 1-3
Short Run Low Grey Emphasis	0.0002 (0.0002–0.0002)	0.0002 (0.0002–0.0002)	0.0002 (0.0002–0.0002)	<0.001	1-2, 1-3
Long Run Low Grey Emphasis	0.01 (0.01–0.02)	0.02 (0.01–0.02)	0.02 (0.01–0.02)	0.006	1-2, 1-3
Long Run High Grey Emphasis	226 k (86 k–342 k)	321 k (231 k–376 k)	346 k (237 k–412 k)	0.005	1-2, 1-3
Run Percentage	0.73 (0.72–0.77)	0.72 (0.72–0.73)	0.72 (0.71–0.72)	0.001	1-2, 1-3

**Table 4:** Comparison of Tc-99m DMSA -based Radiomic Features: Morphological Features.

Radiomic Feature	Group 1 (DM + HTN)	Group 2 (HTN)	Group 3 (Non-DM/Non-HTN CKD)	*p* Value	Post Hoc Analysis (*p* < 0.05)
Compacity	197.41 (188.66–212.02)	192.26 (178.57–201.53)	187.60 (178.68–196.35)	0.038	1-3
Spherical Disproportion	7.01 (6.80–7.35)	6.89 (6.56–7.11)	6.77 (6.56–6.99)	0.043	1-3
Asphericity	6.01 (5.80–6.35)	5.89 (5.56–6.11)	5.77 (5.56–5.99)	0.043	1-3
Centre Of Mass Shift	1.14 (0.90–1.31)	1.28 (1.09–1.51)	1.23 (1.12–1.50)	0.037	1-3
Radius Sphere Norm (Centroid)	0.12 (0.09–0.13)	0.13 (0.11–0.15)	0.13 (0.12–0.15)	0.024	1-3
Radius Roi Norm (Centroid)	0.02 (0.02–0.03)	0.03 (0.02–0.03)	0.03 (0.02–0.03)	0.003	1-2, 1-3
Max Int. Coor (Axial Dist)	11.70 (9.59–15.55)	17.48 (12.99–22.37)	18.61 (14.20–23.94)	0.010	1-2, 1-3
Radius Sphere (Axial Dist)	1.23 (1.03–1.67)	1.80 (1.37–2.37)	1.99 (1.44–2.41)	0.009	1-2, 1-3
Radius Roi (Axial Dist)	0.21 (0.18–0.33)	0.33 (0.26–0.45)	0.36 (0.26–0.47)	0.005	1-2, 1-3
Max Int. Coor (Sagittal Dist)	18.39 (14.04–29.84)	23.11 (19.36–26.90)	26.90 (20.50–33.60)	0.010	1-3
Radius Sphere (Sagittal Dist)	1.96 (1.47–3.08)	2.36 (2.01–2.84)	2.78 (2.20–3.30)	0.006	1-3, 2-3
Radius Roi (Sagittal Dist)	0.34 (0.26–0.58)	0.45 (0.38–0.51)	0.53 (0.42–0.64)	0.004	1-3, 2-3

Abbreviations: ROI, region of interest.

**Table 5:** Univariate L2-Penalised Cox Regression: Radiomic Predictors of Time to ≥20% eGFR Decline.

Radiomic Feature	HR (Per SD)	95% CI	*p*	C-Index
Centre of Mass Shift	0.74	0.58–0.94	0.015	0.57
Surface Area ^a^	0.68	0.52–0.88	0.003	0.63
Voxels Counting ^a^	0.67	0.52–0.87	0.003	0.63
RMS Intensity	0.92	0.71–1.18	0.497	0.53

HR = hazard ratio per one SD increase in z-score-standardised feature value; CI = confidence interval; C-index = Harrell’s concordance index. All analyses used L2-penalised (Ridge, λ = 0.1) univariate Cox proportional hazards regression; follow-up endpoint: first occurrence of ≥20% eGFR decline from baseline (n = 44 events in analysed sample; 9 patients excluded due to missing follow-up data). ^a^ Surface Area and Voxels Counting share near-collinear information (Spearman r = 0.96) and should not be interpreted as independent predictors.

## Data Availability

The datasets generated during and/or analysed during the current study are available from the corresponding author on reasonable request.

## Supplementary Materials

The following supporting information can be downloaded at: https://www.mdpi.com/article/10.3390/diagnostics16091351/s1.
