# Supplementary material for: Tc-99m DMSA Radiomics in CKD: Phenotype-Specific Cortical Signatures and a Morphological Predictor of Renal Function Decline
Source: Diagnostics (Basel). 2026 Apr 30;16(9):1351. doi: 10.3390/diagnostics16091351 (PMC13163433; doi:10.3390/diagnostics16091351)
Supplement: Supplementary file 1 [file diagnostics-16-01351-s001.zip › diagnostics-4245934-supplementary.pdf]

INTENSITY BASED FEATURES					
**Radiomic Feature**	Group 1 (DM+HTN)	Group 2 (HTN)	Group 3 (non-DM/non-HTN CKD)	P Value	Post-hoc Analysis (p<0.05)
**Integrated Intensity**	199471 (109509-307559)	293787 (220058-332885)	318366 (270730-362739)	0.001	1-2, 1-3
**Mean Intensity**	60.66 (31.01-89.39)	79.27 (65.85-91.39)	90.46 (72.87-106.34)	<0.001	1-2, 1-3
**Median Intensity**	58.99 (31.11-86.92)	76.91 (63.93-89.83)	88.63 (69.78-103.19)	<0.001	1-2, 1-3
**Minimum Intensity**	41.30 (17.67-57.45)	54.43 (45.90-63.49)	61.17 (49.31-71.62)	<0.001	1-2, 1-3
**Maximum Intensity**	102.53 (56.14-142.21)	135.59 (114.93-157.18)	151.77 (122.40-177.67)	<0.001	1-2, 1-3
**Intensity Variance**	162.69 (48.37-299.96)	275.08 (179.47-379.94)	359.91 (215.35-476.91)	<0.001	1-2, 1-3
**Intensity Skewness**	0.60 (0.46-0.71)	0.54 (0.43-0.65)	0.53 (0.43-0.62)	0.296	-
**Intensity Kurtosis**	-0.11 (-0.31-0.12)	-0.24 (-0.41-0.04)	-0.33 (-0.45--0.16)	0.001	1-3, 2-3
**10th Intensity Percentile**	45.14 (22.63-64.99)	59.90 (49.27-68.78)	68.09 (54.63-79.24)	<0.001	1-2, 1-3
**25th Intensity Percentile**	50.38 (25.96-75.63)	67.17 (55.05-76.60)	76.31 (60.79-88.45)	<0.001	1-3
**75th Intensity Percentile**	68.98 (35.93-100.07)	90.30 (74.82-104.53)	103.05 (82.53-121.07)	<0.001	1-2, 1-3
**90th Intensity Percentile**	78.59 (40.44-112.91)	102.32 (83.99-118.53)	117.85 (94.46-137.04)	<0.001	1-2, 1-3, 2-3
**Standard Deviation**	12.64 (6.91-17.09)	16.52 (13.18-19.35)	18.96 (14.67-21.84)	<0.001	1-2, 1-3
**Intensity IQR**	18.41 (9.81-24.53)	24.06 (18.71-29.19)	27.68 (20.99-31.89)	<0.001	1-2, 1-3

**Intensity Range**	61.22 (35.47-84.76)	81.11 (69.21-93.67)	90.63 (73.09-106.25)	<0.001	1-2, 1-3
**Mean Absolute Deviation**	10.31 (5.61-13.92)	13.55 (10.75-16.11)	15.40 (12.02-17.87)	<0.001	1-2, 1-3
**Robust Mean Abs. Dev.**	7.58 (4.16-10.53)	10.03 (7.90-12.20)	11.45 (8.84-13.30)	<0.001	1-2, 1-3
**Median Absolute Deviation**	10.21 (5.56-13.89)	13.41 (10.61-15.99)	15.26 (11.87-17.79)	<0.001	1-2, 1-3
**Coeff. Of Variation**	0.20 (0.20-0.21)	0.20 (0.20-0.21)	0.20 (0.20-0.21)	0.940	-
**Quartile Coeff. Dispersion**	0.15 (0.14-0.15)	0.15 (0.14-0.16)	0.15 (0.15-0.16)	0.352	-
**Max Histogram Gradient**	435.16 (329.52-496.04)	445.42 (373.78-486.12)	433.50 (389.54-476.90)	0.919	-
**Area Under Curve**	19.17 (9.98-28.18)	25.01 (20.83-28.80)	28.49 (22.98-33.40)	<0.001	1-2, 1-3
**Intensity Based Energy**	3.1M (969k-6.6M)	6.2M (4.0M-7.8M)	7.5M (5.2M-9.6M)	<0.001	1-3
**Root Mean Square Intensity**	61.97 (31.75-91.05)	80.90 (67.29-93.29)	92.46 (74.47-108.58)	<0.001	1-2, 1-3
**Total Lesion Glycolysis**	213.40 (123.95-324.97)	308.01 (234.68-349.11)	333.23 (288.09-383.33)	0.001	1-2, 1-3
**Root Mean Square**	0.03 (0.03-0.03)	0.03 (0.03-0.04)	0.03 (0.03-0.04)	0.062	-
**TEXTURE FEATURES**					
**Grey Level NonUniformity**	629.50 (455.92-717.72)	637.68 (539.99-700.26)	615.09 (558.41-683.20)	0.940	-
**Short Runs Emphasis**	0.79 (0.78-0.86)	0.78 (0.76-0.79)	0.77 (0.76-0.78)	<0.001	1-2, 1-3
**Long Runs Emphasis**	55.38 (21.07-83.72)	78.49 (56.63-92.02)	84.55 (57.93-100.77)	0.005	1-2, 1-3
**Low Grey Level Run Emphasis**	0.0002 (0.0002-0.0003)	0.0002 (0.0002-0.0002)	0.0002 (0.0002-0.0002)	0.001	1-2, 1-3

**Short Run Low Grey Emphasis**	0.0002 (0.0002-0.0002)	0.0002 (0.0002-0.0002)	0.0002 (0.0002-0.0002)	<0.001	1-2, 1-3
**Short Run High Grey Emphasis**	3208 (3118-3271)	3172 (3123-3215)	3156 (3100-3202)	0.124	-
**Long Run Low Grey Emphasis**	0.01 (0.01-0.02)	0.02 (0.01-0.02)	0.02 (0.01-0.02)	0.006	1-2, 1-3
**Long Run High Grey Emphasis**	226k (86k-342k)	321k (231k-376k)	346k (237k-412k)	0.005	1-2, 1-3
**Run Length NonUniformity**	655.75 (585.83-745.16)	632.38 (547.16-681.66)	602.80 (543.18-663.87)	0.171	-
**Run Percentage**	0.73 (0.72-0.77)	0.72 (0.72-0.73)	0.72 (0.71-0.72)	0.001	1-2, 1-3
**Run Length Variance**	25.16 (12.79-37.39)	32.39 (26.01-36.26)	31.70 (25.67-36.30)	0.079	-
**Run Entropy**	1.18 (1.14-1.36)	1.20 (1.16-1.24)	1.20 (1.18-1.23)	0.484	-
**Large Zone Emphasis**	723k (190k-985k)	771k (553k-946k)	726k (588k-903k)	0.741	-
**Small Zone High Grey Emphasis**	0.007 (0.004-1432)	0.005 (0.004-0.008)	0.006 (0.005-0.007)	0.281	-
**Large Zone Low Grey Emphasis**	176.52 (46.42-240.50)	188.41 (135.22-231.08)	177.25 (143.67-220.54)	0.741	-
**Large Zone High Grey Emphasis**	2.9B (0.7B-4.0B)	3.1B (2.2B-3.8B)	2.9B (2.4B-3.7B)	0.741	-
**Zone Percentage**	0.0013 (0.0010-0.0631)	0.0011 (0.0010-0.0014)	0.0012 (0.0011-0.0013)	0.255	-
MORPHOLOGICAL FEATURES					
**Voxel Count**	1731 (1347-1982)	1756 (1486-1930)	1684 (1534-1887)	0.842	-
**Surface Area**	147.95 (120.25-174.97)	153.82 (130.49-169.86)	146.87 (134.37-164.87)	0.764	-
**Sphere Diameter**	19.56 (18.64-20.24)	19.53 (18.50-19.97)	19.17 (18.52-19.83)	0.765	-

**Compacity**	197.41 (188.66-212.02)	192.26 (178.57-201.53)	187.60 (178.68-196.35)	0.038	1-3
**Compactness 1**	0.003 (0.003-0.003)	0.003 (0.003-0.003)	0.003 (0.003-0.003)	0.089	-
**Compactness 2**	0.003 (0.003-0.003)	0.003 (0.003-0.004)	0.003 (0.003-0.004)	0.124	-
**Spherical Disproportion**	7.01 (6.80-7.35)	6.89 (6.56-7.11)	6.77 (6.56-6.99)	0.043	1-3
**Sphericity**	0.14 (0.14-0.15)	0.15 (0.14-0.15)	0.15 (0.14-0.15)	0.074	-
**Asphericity**	6.01 (5.80-6.35)	5.89 (5.56-6.11)	5.77 (5.56-5.99)	0.043	1-3
**Centre Of Mass Shift**	1.14 (0.90-1.31)	1.28 (1.09-1.51)	1.23 (1.12-1.50)	0.037	1-3
**Max Int. Roi Centroid Dist.**	17.34 (15.04-20.29)	16.01 (12.98-18.94)	15.01 (12.44-18.40)	0.174	-
**Radius Sphere Norm (MaxInt)**	1.85 (1.55-2.12)	1.61 (1.37-1.99)	1.58 (1.28-1.91)	0.136	-
**Radius Roi Norm (MaxInt)**	0.33 (0.29-0.38)	0.30 (0.26-0.38)	0.30 (0.24-0.36)	0.386	-
**Radius Sphere Norm (Centroid)**	0.12 (0.09-0.13)	0.13 (0.11-0.15)	0.13 (0.12-0.15)	0.024	1-3
**Radius Roi Norm (Centroid)**	0.02 (0.02-0.03)	0.03 (0.02-0.03)	0.03 (0.02-0.03)	0.003	1-2, 1-3
**Max Int. Coor (Axial Dist)**	11.70 (9.59-15.55)	17.48 (12.99-22.37)	18.61 (14.20-23.94)	0.010	1-2, 1-3
**Radius Sphere (Axial Dist)**	1.23 (1.03-1.67)	1.80 (1.37-2.37)	1.99 (1.44-2.41)	0.009	1-2, 1-3
**Radius Roi (Axial Dist)**	0.21 (0.18-0.33)	0.33 (0.26-0.45)	0.36 (0.26-0.47)	0.005	1-2, 1-3
**Max Int. Coor (Sagittal Dist)**	18.39 (14.04-29.84)	23.11 (19.36-26.90)	26.90 (20.50-33.60)	0.010	1-3

**Radius Sphere (Sagittal Dist)**	1.96 (1.47-3.08)	2.36 (2.01-2.84)	2.78 (2.20-3.30)	0.006	1-3, 2-3
**Radius Roi (Sagittal Dist)**	0.34 (0.26-0.58)	0.45 (0.38-0.51)	0.53 (0.42-0.64)	0.004	1-3, 2-3
